# Modulated Degradation of Polylactic Acid Electrospun Coating on WE43 Stents

**DOI:** 10.3390/polym17111510

**Published:** 2025-05-28

**Authors:** Mariana Macías-Naranjo, Marilena Antunes-Ricardo, Christopher Moreno González, Andrea Noelia De la Peña Aguirre, Ciro A. Rodríguez, Erika García-López, Elisa Vazquez-Lepe

**Affiliations:** 1 Tecnologico de Monterrey, School of Engineering and Sciences, Ave. Eugenio Garza Sada 2501 Sur, Monterrey 64700, N.L, Mexico; a01280232@tec.mx (M.M.-N.); a00829580@tec.mx (C.M.G.); a00829071@tec.mx (A.N.D.l.P.A.); ciro.rodriguez@tec.mx (C.A.R.); 2 Tecnologico de Monterrey, The Institute for Obesity Research, Ave. Eugenio Garza Sada 2501 Sur, Monterrey 64700, N.L, Mexico; marilena.antunes@tec.mx

**Keywords:** coronary stent, magnesium alloy, PLA, corrosion resistance, biocompatibility

## Abstract

Magnesium-based coronary stents have gained significant interest due to their excellent biocompatibility, biodegradability, and mechanical properties. However, a key limitation of magnesium in biomedical applications is its low corrosion resistance, which compromises its structural integrity and mechanical strength over time. Polymeric coatings can overcome this challenge, enhancing magnesium-based implants’ corrosion resistance and overall performance. This study applied a polylactic acid (PLA) nanofiber coating to WE43 magnesium (Mg) stents via electrospinning to reduce their corrosion rate. Both uncoated and coated stents underwent in vitro immersion tests in Hank’s solution for 1, 3, 7, and 14 days. The effectiveness of the PLA coating was evaluated through morphological analysis, chemical composition assessment, corrosion behavior (weight change), magnesium ion release, and in vitro biocompatibility. The corrosion observed in the uncoated WE43 stents indicates that protective coatings are necessary to regulate degradation rates over extended implantation periods. The results demonstrated that coated stents exhibited improved performance, maintaining the integrity of the PLA coating for up to 14 days. The coated stents demonstrated reduced surface damage and lower weight loss resulting from lower magnesium release. In our study, the coated stents demonstrated a reduced corrosion rate (0.216 ± 0.013 mm/year) compared with the uncoated stents (0.312 ± 0.010 mm/year), both after 14 days. Additionally, in vitro biocompatibility results confirmed the non-toxic nature of PLA-coated stents, which enhances cellular proliferation and contributes to a more favorable environment for vascular healing. These findings suggest that PLA coatings can effectively prolong the functional durability of WE43 Mg stents, offering a promising solution for enhancing the performance of biodegradable stents in cardiovascular applications.

## 1. Introduction

Nowadays, cardiovascular disease continues to be the leading cause of death worldwide, accounting for more than 17.9 million deaths each year [1]. In 2020, this number increased to 19.05 million [2]. The implementation of stents into the vessel is one of the most widely accepted ways of treating coronary artery disease (CAD) [3,4]. Therefore, this field of research is constantly progressing, moving from bare-metal stents to drug-eluting stents and, more recently, exploring bioresorbable stents [4]. Despite significant advances in the development of stents, they still have disadvantages, including in-stent restenosis, stent fracture, and the risk of thrombosis due to body rejection. Consequently, many authors continue to develop a coronary stent that will be safe, effective, durable, affordable, and compatible with patients.

Metal implants have been preferred due to their long-term effectiveness, stability, high strength, and excellent ductility [5]. However, hazardous metal ions caused by corrosion and wear can cause inflammation, cell apoptosis, and other tissue reactions [5]. This led to conventional bare metallic stents being replaced due to their restenosis rate and the need for a second intervention. For this reason, in recent years, biodegradable stents (BMs) have gained significant attention due to their unique degradation properties and robust mechanical traits [2]. Biodegradable stents must have high biocompatibility to reduce inflammation and allergic reactions, appropriate degradation behavior providing 3–6 months of mechanical strength followed by complete degradation within 12–24 months, and they must have strong mechanical properties, providing stronger radial strength and compression resistance [2,6]. Among the biodegradable materials, magnesium (Mg) and its alloys have generated interest due to their exceptional properties in the biomedical sector, such as cardiovascular interventions [7]. This is due to their outstanding advantages, such as biodegradability, low density, excellent moldability, mechanical properties, and biocompatibility [3,8,9]. Furthermore, Mg is a bioactive substance that degrades rapidly and naturally in the body (in 1–4 months) [5], being an essential element [10] and thus eliminating the need for surgery to remove the implant [11]. However, a critical disadvantage of magnesium for engineering applications is its low corrosion resistance, especially under physiological conditions (pH 7.4–7.6) [12], affecting its integrity and mechanical properties and reducing the time for the implant to fulfill its purpose before being degraded [8,9].

Among the various alloys of magnesium, the WE43 Mg alloy meets the requirements for implant applications by reducing long-term thermal risks due to its anti-proliferative properties and its reduced restenosis rate compared to conventional metallic stents, such as stainless steel [10]. The advantage of the WE43 alloy lies in its enhanced corrosion resistance compared to pure magnesium, which is attributed to the presence of rare earth elements in its composition [9]. Moreover, this alloy is aluminum-free, improving essential requirements without the controversial aspects of aluminum, such as its potential neurotoxicity and carcinogenicity [9,11]. Alloys like WE43 contain rare-earth elements, which are considered promising materials for biomedical applications due to their improved mechanical properties and biocompatibility. However, further research is still needed to assess their properties for specific application requirements thoroughly [11].

Polymeric coatings have been utilized as a surface modification method to enhance Mg-based implants’ corrosion resistance and overall performance [13]. Various natural (fibrin, alginate, chitosan) and synthetic polymers (polylactic acid (PLA), poly(glycolic acid) (PGA), poly(lactide-co-glycolide) (PLGA), and polycaprolactone (PCL)) have been used to protect Mg from corrosion [5,14]. Table 1 shows that polylactic acid (PLA) coatings have been widely studied for biomedical applications, including cardiovascular and orthopedic applications, on Mg-based substrates due to their biocompatibility, biodegradability, and low degradation rate (degradation by hydrolysis), resulting in non-toxic products [5,15]. Abdal-hay et al. coated AM50 magnesium discs with PLA using two different techniques, electrospinning and dip coating, to enhance their degradation resistance. They evaluated the corrosion resistance of coated and uncoated samples through immersion tests in a physiological solution and electrochemical assays, demonstrating that both coatings improved the degradation resistance of magnesium. Cytocompatibility tests with MC3T3-E1 osteoblast cells showed that both coatings enhanced cell adhesion and proliferation, with the electrospun nanofiber membranes performing better, highlighting electrospinning as a promising method for Mg-based materials in orthopedic and cardiovascular applications [16]. Peng et al. studied the effect of a poly-L-lactic acid (PLLA) coating on AZ31 squares via a two-step method: fluorine conversion coating and PLLA coating via the ultrasonic atomization method. The composite coating demonstrated a higher corrosion resistance and better endothelization and hemocompatibility, providing a promising candidate for cardiovascular stents [17]. Kumar et al. coated Mg–Zn–Mn plates with PLA using the sol-gel technique for temporary implants. Through in vitro corrosion studies, PLA reduced the corrosion rate by 73% compared to the bare alloy.

Additionally, cytotoxicity tests with MG-63 cells demonstrated the material’s biocompatibility, with the effectiveness of the PLA coating attributed to the synergistic effect of physical and chemical interactions with the magnesium alloy [18]. Mardani et al. deposited a PLA coating via 3D printing on AM60 magnesium plates. PLA increased the corrosion resistance compared to the Mg substrate after 24 h of exposure [19]. Liu et al. prepared fluorinated treatment coatings (MgF2), polylactic acid (PLA) coatings, and composite coatings (MgF_2_/PLA) for Mg-2.2Zn-0.3Ca alloy wires to enhance their corrosion properties. They obtained the most protective effect with a composite MgF_2_/PLA coating, but the PLA coating also showed good long-term anticorrosion properties [20].

**Table 1:** Literature review of polylactic acid (PLA) used on magnesium alloy substrates for biomedical applications.

Substrate Magnesium Alloy	Substrate Geometry	Additional Pre-Treatment Coating Material	Polymer Coating Method	Application	Ref.
AZ91	-	- PLA	Spin coating	Orthopedic	[21]
AM50	Disks	- PLA	Dip coating and Electrospinning	Orthopedic and cardiovascular	[16]
AZ31	Plates	Micro-arc oxidation (MAO) PLA	Dip coating	Orthopedic	[22]
AZ31	Disks	Electro-deposition, alkali-heat-treatment and sol-gel (TiO_3_) HA, HA/PLA, and HA/CaTiO_3_/TiO_2_/PLA	Dip coating	Orthopedic	[23]
Mg−Nd−Zn−Zr (JDBM)	Disks	Conversion coating (hydrofluoric acid) PLA and dicalcium phosphate dihydrate (brushite, DCPD)	Dip coating	Orthopedic	[24]
Pure Mg	Rods	- PLLA	Dip coating	Orthopedic	[25]
Mg-Ca	Squares	- Poly-L-lactic acid (PLLA), åkermanite (AKT; Ca_2_MgSi_2_O_7_) and doxycycline (DOXY)	Electrospinning	Orthopedic	[26]
AZ31	Squares	- PLA	Electrospinning	Cardiovascular	[27]
AZ31	Squares	Fluorine conversion coating PLLA	Ultrasonic atomization	Cardiovascular stent	[17]
AZ31B and AZ31	Disks and stents	Hydrofluoric acid (HA), poly(butyl acrylate (PBA), and poly(d,L-lactide) PDLLA	Dip coating and Ultrasonic atomization	Coronary stent	[28]
AM50	Plates	- PLA	Fused Deposition Modeling (FDM)	Orthopedic	[29]
Mg–Zn–Mn	Squares	Alkali solution (NaOH) PLA	Sol-gel	Orthopedic	[18]
Mg–2.2Zn–0.3Ca	Wires	Fluoride passivation (MgF2) PLA	Dip coating	General implants	[20]
AM60	Plates	Glue stick of cyanoacrylate PLA	Fused filament fabrication modeling (FFF)	General implants	[19]
WE43	Squares	CaP coating PLA	Dip coating	Orthopedic	[30]
WE43	Coronary stents	Acid pickling PLA	Electrospinning	Coronary stent	This study

According to Table 1, most studies focus on flat or simple geometries, and do not address the challenges of coating complex three-dimensional structures such as coronary stents. This represents a limitation in current approaches, as uniform coatings on intricate geometries are critical for clinical performance. Despite the progress in polymer-based coatings for magnesium stents, several limitations persist in current approaches. Dip coating often results in unevenness, especially on complex geometries; air bubbles can become trapped in certain areas of the surface that may hinder endothelialization or delay degradation [31,32]. Spray coating can suffer from low control over roughness and limited control over coating thickness, especially on complex geometries such as stents [33]. Moreover, both techniques may leave defects that allow localized corrosion to initiate. While some studies report promising results, there is often a lack of consistency in coating uniformity and long-term degradation control. These challenges underline the need for alternative strategies. In this context, electrospinning emerges as a promising technique due to its ability to produce uniform, nanostructured coatings with enhanced surface coverage and tunable degradation profiles, which supports the novelty and relevance of the present study [34].

In this work, a polylactic acid (PLA) nanofiber coating was applied via electrospinning to mitigate the corrosion rate of WE43 Mg stents while preserving their structural integrity. Uncoated and coated stents were subjected to in vitro immersion tests in Hank’s solution for 1, 3, 7, and 14 days at 37 °C, with pH monitoring to ensure experimental control. The effect of the coating was evaluated through morphological analysis, chemical composition assessment, corrosion behavior (weight change), magnesium ion release, and in vitro cell viability. The PLA coating is expected to enhance the functional lifespan of Mg-based stents, presenting a promising approach for their use in cardiovascular applications. The novelty of this study lies in the application of PLA electrospun coatings on complex stent geometries of biodegradable materials such as magnesium. In the current literature, the development of effective protective film on magnesium alloys remains a challenge due to the balance between corrosion control and the promotion of cell proliferation, which can influence healing or lead to potential complications. Additionally, the use of electrospinning to deposit a protective mat on metallic substrates is of great interest, as it offers precise control over fiber morphology, surface coverage, and controlled degradation behavior.

## 2. Materials and Methods

### 2.1. Materials and Samples Geometry

**Figure 1:**
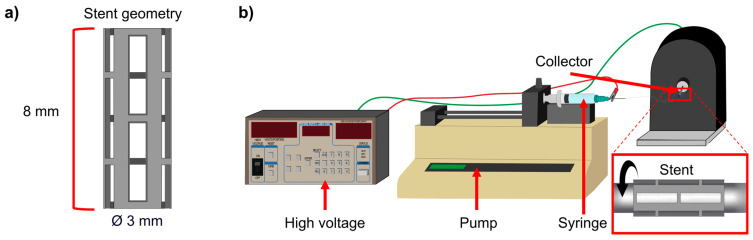
(**a**) Sample geometry and (**b**) electrospinning setup.

WE43 magnesium alloy minitubes with an outer diameter of 3 mm and a wall thickness of 0.25 mm (Complex Materials, Eindhoven, The Netherlands) were laser-cut using a 4-axis laser cutting machine (Preco, MedPro ST2000, St. Croix, VI, USA). Samples were cut using an average laser power of 200 W, a frequency of 1250 Hz, a feed rate of 400 mm/min, and a pulse on of 0.000168 s. Figure 1a shows the stent geometry with its dimensions. For the coating solution, polylactic acid (PLA) pellets were utilized as the solute (MW = 231,000 g/mol, Sigma-Aldrich, GF45989881, Burlington, NJ, USA) dissolved in a solvent mixture of 2:1 chloroform/acetone. Chloroform (CLF) (CHCl_3_) (67-66-3) and acetone (AC) (C_3_H_6_O) (67-64-1) were provided by CTR Scientific (Monterrey, Mexico).

### 2.2. Acid Pickling

Acid pickling was applied to assure surface quality after laser cutting and to reduce the cutting edge’s surface roughness. WE43 Mg stents were ultrasonically cleaned with a 70% *v*/*v* ethanol/distilled water solution for 5 min. Subsequently, WE43 Mg stents were immersed in 20 mL of the etchant solution (10% *v*/*v* nitric acid/ethanol) for different amounts of time (1, 1.5, and 2 min). Finally, samples were washed using a 70% ethanol/distilled water solution for 5 min and dried with compressed air. After completing the tests, the three samples were examined morphologically under an Olympus SZH stereo microscope (Olympus, Hachiōji, Japan), where images were taken, and comparisons were made to determine the optimal parameters. A one-way ANOVA was performed to statistically evaluate the effect of immersion time on strut thickness.

### 2.3. Electrospinning

A high-voltage power supply (Stanford Research Systems, Inc., PS375, Sunnyvale, CA, USA) generated an electric field of 15 kV. A pump (KD Scientific, KDS-200, Holliston, MA, USA) fed the PLA solution with a flow rate of 0.05 mL/min through a 10 mL glass syringe through a metallic needle (14 Ga) located 15 cm from the sample. Figure 1b shows a stainless steel tube set as the collector to rotate the sample, connected to a motor that controls the rotational speed. The spinning time for all experiments was 2 min. All experiments were performed at room temperature (22 ± 1 °C) and relative humidity (53 ± 3%).

For the electrospinning solution, PLA pellets were dried in an oven at 50 °C for 12 h. In a previous study, solvents were analyzed, and their concentration was investigated through rheological testing. Therefore, 10% *w*/*v* of PLA was dissolved in a 2:1 (*v*/*v*) solvent mixture of chloroform and acetone by magnetic stirring at room temperature until complete dissolution.

Fiber characterization was analyzed via scanning electron microscope (SEM) (Zeiss, EVOMA25, Jena, Germany) to observe surface uniformity. ImageJ software (Version 1.53t, National Institutes of Health, Bethesda, MD, USA) measured Fiber diameters. One hundred measurements were taken for each test to calculate the average diameter.

### 2.4. Immersion Test

The immersion test was conducted according to ASTM-G31-72 in Hank’s Balanced Salts Solution (HBSS—Hank’s solution) provided by CTR Scientific (Monterrey, MX). Uncoated stents and coated stents (PLA electrospun fibers) were cast in 22 mL (according to the exposed surface) of Hank’s solution at 37 °C using a water bath for 1, 3, 7, and 14 days (1d, 3d, 7d, and 14d) without agitation. The solution was replaced every 24 h to maintain pH ~ 7.4. After the immersion test, the samples were removed and kept in a desiccator. Two samples were taken for each casting. Stents were weighed before and after immersion according to the exposure days, and pH measurements of the solution were taken daily to monitor any changes.

The surface morphology and corrosion were evaluated using scanning electron microscopy (SEM) equipped with an energy-dispersive spectrometer (EDS) attachment. Samples were covered with a thin layer of gold (1 nm) by sputtering to improve the low contrast due to the polymeric thin film deposited. ICPMS (inductively coupled plasma mass spectrometry) metal analysis was performed on an Agilent 7500ce ICPMS instrument (Agilent Technologies, Santa Clara, CA, USA) to determine accurate concentrations of Mg^2+^ released into the immersion solution after testing. Quantitative analysis of the cracked surface area was performed along the strut (uncoated stents) using ImageJ software (Version 1.53t, National Institutes of Health, Bethesda, MD, USA), modifying threshold in the image to highlight crack zones and converting the image to binary.

After characterization, the stents were dipped in 20 mL of 10% *v*/*v* nitric acid/ethanol for 20 s to remove residue from Hank’s solution. Samples were ultrasonically washed using a 70% ethanol/distilled water solution for 5 min and dried with compressed air. Finally, surface morphology and weight were measured again to compare with the previous results.

### 2.5. In Vitro Biocompatibility

Human dermal fibroblasts (HDFa) obtained from the American Type Culture Collection (ATCC, Manassas, VA, USA) were cultured in Petri dishes with Dulbecco’s Modified Eagle’s Medium (DMEM) (Gibco©, Grand Island, NE, USA) supplemented with 5% fetal bovine serum (FBS) (Gibco©, Grand Island, USA) at 37 °C in a 5% CO_2_ incubator (Thermofisher, BB150–2TCS, Waltham, MA, USA). Cell media were changed every 48 h until 80% confluence was achieved.

Stents were sterilized with ultraviolet (UVC) light (at 260 nm) for 20 min. After this, stents were placed in 12-well plate, and cells were seeded at a concentration of 2.25 × 10^5^ cells per well in supplemented medium (DMEM with 5% FBS) and cultured at 37 °C and 5% CO_2_. Cells with stents were incubated for 72 h at 37 °C and 5% CO_2_ without agitation. Cells without stents were used as the control group.

The cell viability was measured according to the manufacturer’s instructions of the Cell Titer 96 Aqueous One Solution Kit^®^ (Promega Corporation, Madison, WI, USA). Then, 100 μL of Cell Titer solution was added to the samples, incubated for 75 min, and absorbance was measured at 490 nm on a 96-well plate reader (Synergy HT, Bio-Tek, Winooski, VT, USA). Cell viability was determined according to Equation (1):

(1) $$Cell\;viability\;(\%)=\frac{Abs\;treated\;cells}{Abs\;control\;cells}\times 100$$

where Abs treated cells corresponds to the average absorbance of cells in direct contact with the tested stents, whereas Abs control cells correspond to the average absorbance of untreated cells (control). All tests were performed in triplicate. The statistical significance was determined using one-way ANOVA followed by Tukey’s test. A *p*-value of < 0.05 was considered to represent a significant difference between values.

To assess cell proliferation in stents, cells were cultured as described above. After 72 h of incubation with the stents, cells were washed with phosphate-buffered saline (PBS) to remove unattached cells. Then, cells were fixed using cool methanol for 10 min. After this, methanol was removed, and samples were washed with PBS. DAPI dye (4′,6-diamidino-2-phenylindole) (D9542, Sigma-Aldrich, Burlington, VT, USA) was added (300 nM in PBS) to each sample and incubated in dark conditions for 10 min. After this time, treated wells were washed twice with PBS, and finally, cells were observed by fluorescent microscopy on an EVOSFLc inverted fluorescence microscope (Thermo Fisher Scientific, Waltham, MA, USA) using a fluorescence filter (DAPI (352–477 nm)).

Samples were rinsed with PBS, and a dehydration process was carried out using a graded ethanol series (10%−100% ethanol). The samples were then dried and sputter-coated with gold. Surface morphology and cell attachment were examined using SEM operated at 10 kV.

## 3. Results and Discussion

### 3.1. Stent Surface Preparation

**Figure 2:**
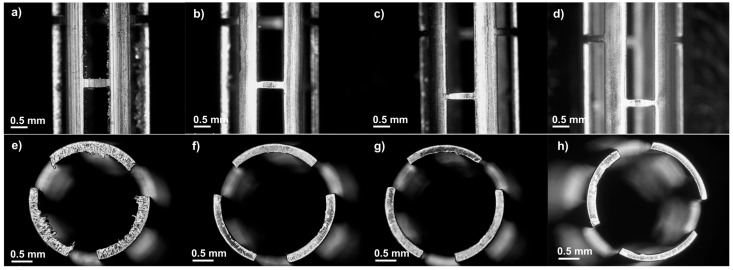
Optical images at lateral (25×) and top view (30×): (**a**,**e**) before acid pickling, (**b**,**f**) after 1 min, (**c**,**g**) after 1.5 min, and (**d**,**h**) after 2 min.

Figure 2 illustrates the WE43 Mg stents before and after acid pickling for different lengths of time. Figure 2a,e show the stent after laser cutting. Some defects, such as irregular cut edges and dross particles, were observed after laser cutting. Three durations were tested to determine the optimal etching time: 1, 1.5, and 2 min. After pickling, the etched stents exhibited a cleaner surface, as the amount of material removed increased with longer etching times [35]. The initial strut thickness of the stent was 247.6 ± 2.5 μm. Strut thickness is a critical factor, as it affects the structural integrity of the material during biodegradation tests. Premature collapse of the struts may lead to mechanical failure of the stent, increasing the risk of restenosis [2]. Analyzing the thickness of the struts after 1 min, the strut measured 204.4 ± 11.3 μm; after 1.5 min, the strut decreased to 191.8 ± 2.6 μm, and compared to the 2 min strut, which measured 189.2 ± 23.1 μm, there was a reduction of 23.58% with the initial strut thickness being the highest material removal. A one-way ANOVA was performed to statistically evaluate the effect of immersion time on strut thickness. The results showed a statistically significant difference between the groups, with a p-value of 0.00001, confirming that the immersion time has a significant influence on the strut thickness. We decided to select an etching time of 1 min for all the stents in the experiment because, according to our preliminary studies, less time was ineffective. Meanwhile an etching time of 1 min resulted in a more uniform strut thickness and smoother cut edges without damage, as is illustrated in Figure 2g. Nwaogu et al. studied the effects of three acids (sulfuric acid, nitric acid, and phosphoric acid) on the corrosion resistance of AZ31 Mg alloy. They concluded that nitric and phosphoric acids could achieve good corrosion resistance (less than 1 mm/year) [36].

### 3.2. Electropun Fibers Production

**Figure 3:**
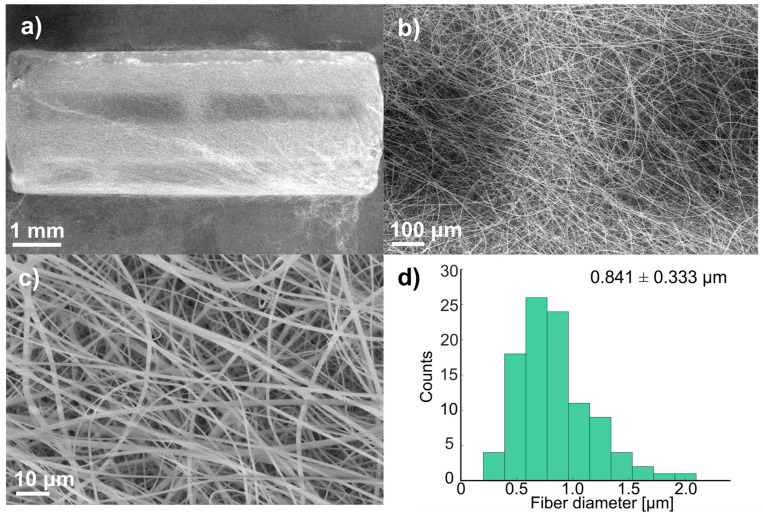
SEM micrographs of coated stent before immersion: (**a**) 15×, (**b**) 25×, (**c**) 1000×, and (**d**) fiber distribution (100 measurements).

Figure 3 shows SEM micrographs and the fiber diameter distribution at three magnifications (15×, 25×, and 1000×) of the stents obtained to analyze the uniformity of deposition and the average fiber diameter. It was observed that free-beaded fibers were formed, showing uniformity along the stents. The fibers were measured at a magnification of 5000×; most fiber diameters were in the range of 0.743–0.958 µm. The average fiber diameter of all the stents was 0.841 ± 0.333 µm. Scaffolds with fibers smaller than 1 µm triggered a lower activation of the coagulation cascade and exhibited reduced platelet adhesion compared to scaffolds made of larger fibers [37].

Also, it is essential to control the thickness of the polymer coating for this application because, as the coating gets thicker, it shows superior anticorrosion properties [7]. Zhao et al. used a thickness of 15 μm to avoid the risk of restenosis and thrombosis due to an excessively thick coating [7]. In this study, the polymer coating thickness was around 23 ± 4 μm, which resulted from the collection time and flow rate.

### 3.3. Degradation Test

**Figure 4:**
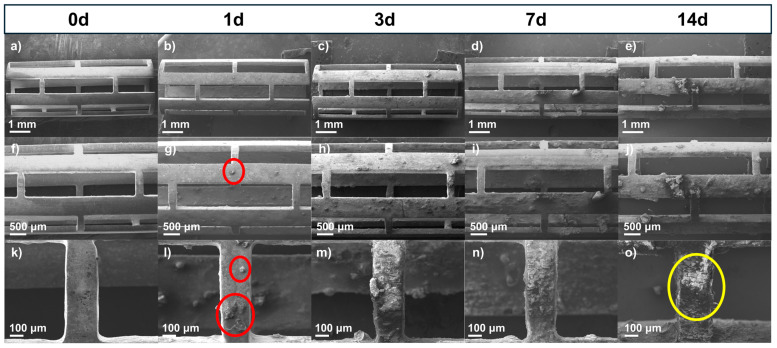
SEM micrographs of uncoated stents before and after immersion test: (**a**–**e**) 15×, (**f**–**j**) 25×, and (**k**–**o**) 100×.

WE43 Mg stents (both uncoated and coated with PLA fibers) were immersed in Hank’s solution at 37 °C for 1, 3, 7, and 14 days to study their dissolution in body fluids, constituting a primary characteristic of biomedical magnesium alloys [38]. Surface analysis of corroded samples demonstrated differences in the morphology of corrosion products between the uncoated and coated stents. Figure 4 shows SEM micrographs of the uncoated stents with different immersion times in the degradation test at three different magnifications (15×, 25×, and 100×). Figure 4a, Figure 4f, and Figure 4k show the stent before being subjected to the degradation test. The surface appeared uniform, with some marks from the material. However, after 1 day (Figure 4b, Figure 4g, and Figure 4l), the surface began to show particles (red circles) formed from Hank’s solution. The size of the formed particles grew as the days passed in the immersion test. The corrosion product size was less than 100 μm; however, after 7 days (Figure 4d, Figure 4i, and Figure 4n) or 14 days (Figure 4e, Figure 4j, and Figure 4o), some exceeded 500 μm, forming a film on the stent. These particles were observed to be volcano-like structures (yellow circle) resulting from hydrogen gas evolution [39]. Another observation is that as the immersion time increased, a higher crack density appeared, indicating pitting corrosion of the WE43 because micro-galvanic corrosion was present between the magnesium matrix and the intermetallic precipitates [40,41]. Figure S1 presents SEM micrographs of sections of the struts from the uncoated WE43 stents after immersion in Hank’s solution for 1, 3, 7, and 14 days (Figure S1a–d), along with the corresponding binary images generated through threshold analysis using ImageJ (Figure S1e–h). A progressive increase in surface cracking was observed over time. Crack density was quantified by calculating the area fraction of the cracked regions, resulting in approximately 12% at day 1, 36% at day 3, 47% at day 7, and 63% at day 14. These results confirm the progression of localized corrosion as immersion time increases.

**Figure 5:**
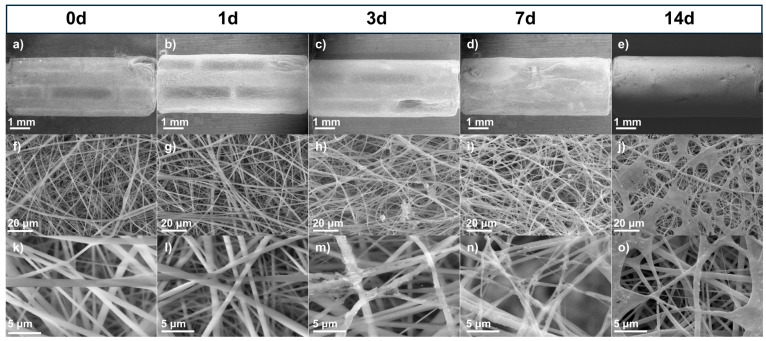
SEM micrographs of coated stents before and after immersion test: (**a**–**e**) 15×, (**f**–**j**) 1000×, and (**k**–**o**) 5000×.

Figure 5 shows the stents coated with PLA fibers to protect the surface from magnesium (Mg). Figure 5a, Figure 5f, and Figure 5k show the stent before being subjected to the degradation test. The surface was uniform, with bead-free fibers. Figure 5b, Figure 5g, and Figure 5l show the micrographs after 1 day, which look similar to the fibers before degradation. However, after 3 days (Figure 5c, Figure 5h, and Figure 5m), the surface began to show particles forming at around 20 μm from Hank’s solution. After 7 days (Figure 5d, Figure 5i, and Figure 5n), particles were seen along the fiber, and after 14 days (Figure 5e, Figure 5j, and Figure 5o), the coating looked like spider webs, a product of Hank’s solution. In Figure 5e, the stent is shown with the fiber coating, and although it remains, crystallization of the salt deposition can be seen. The fibers protected the magnesium surface, but these structures offered a much larger surface to contact with Hank’s solution, which could induce more precipitates [16]. An additional coating technique such as dip coating could be used to produce a film to improve protection. Nevertheless, the fiber coating has a greater surface roughness than other techniques, and for cardiovascular applications, this helps with cell attachment and migration on the implant surfaces [16].

**Figure 6:**
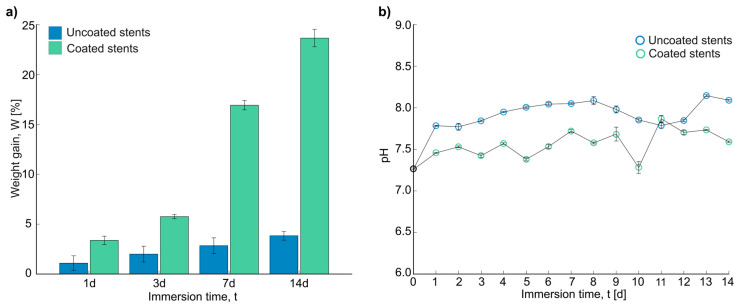
Results after immersion test: (**a**) weight gain in percentage of stents, and (**b**) pH measurements of Hank’s solution for 14 days.

Figure 6a shows the weight gain obtained after the immersion test in Hank’s solution. The corrosion products remained on the samples, which increased their weight. A trend was observed in the uncoated and coated stents: the longer the time in the immersion test, the greater the weight gain. There was a significant difference in weight change between the uncoated and coated stents after 7 days and 14 days of immersion, resulting in the most substantial weight change. As seen in the SEM images, the corrosion products increased in weight. Xue et al. observed white spider-net-like corrosion products on their samples from the PBS solution, and the products formed could have caused the weight increase [41]. In general, there was a higher increase in weight on the coated stents, but this could be because fibers trapped the corrosion products.

The number of electrons and compounds in the solution was measured through pH measurements. Figure 6b shows the final measurements for the uncoated and coated stents over 14 days. The initial pH of all the experiments was 7.24 ± 0.078. As can be seen, the uncoated stents showed a higher final pH, with an average of 7.945 ± 0.129 compared with that of the coated stents, which was 7.575 ± 0.158. According to Liu et al., the pH can be raised due to the presence of many electrons, which promotes the formation of Mg(OH)_2_. This could help to form a Mg(OH)_2_ film, protecting the surface and inhibiting corrosion to some extent [38]. According to Nachtsheim et al., pH values above 8.5 and up to 11.5 could form a protective oxide or hydroxide layer. In the case of magnesium hydroxide dissolution, this could be a strong indicator of severe corrosion [9]. After 5 days of immersion, the uncoated stents had a pH exceeding 8. On the other hand, the maximum pH value for the coated stents was 7.87, indicating mild corrosion. Zhao et al. also obtained the highest pH values with the uncoated Mg samples, indicating the fastest corrosion rate [7].

Various reactions have been studied regarding the effect of aggressive ions on the biodegradable mechanisms of the WE43 Mg alloy [10]. The following anodic reactions can describe the dissolution of Mg [9,10,39]:

(2) $$Mg\left(s\right)\to {Mg}^{2+}\left(aq\right)+2e^{-}$$

(3) $$2H_{2}O+2e^{-}\to H_{2}+2OH^{-}(aq)$$

(4) $${Mg}^{2+}\left(aq\right)+2OH^{-}(aq)\to Mg(OH{)}_{2}(s)$$

The Mg^2+^ is mainly in the form of MgO. The equilibrium between magnesium oxide and hydroxide can be as follows [9]:

(5) $$MgO+2OH^{-}↔Mg(OH{)}_{2}$$

When an oxide film of magnesium hydroxide is formed on the surface, this can slow corrosion. However, when the sample is exposed to a physiological environment with high Cl-levels, Mg(OH)_2_ reacts with Cl^−^ to form highly soluble magnesium chloride [8]. The following reactions explain this behavior [8]:

(6) $$Mg\left(s\right)+2Cl^{-}\left(aq\right)\to MgCl_{2}$$

(7) $$Mg(OH{)}_{2}\left(s\right)+2Cl^{-}\left(aq\right)\to MgCl_{2}$$

**Figure 7:**
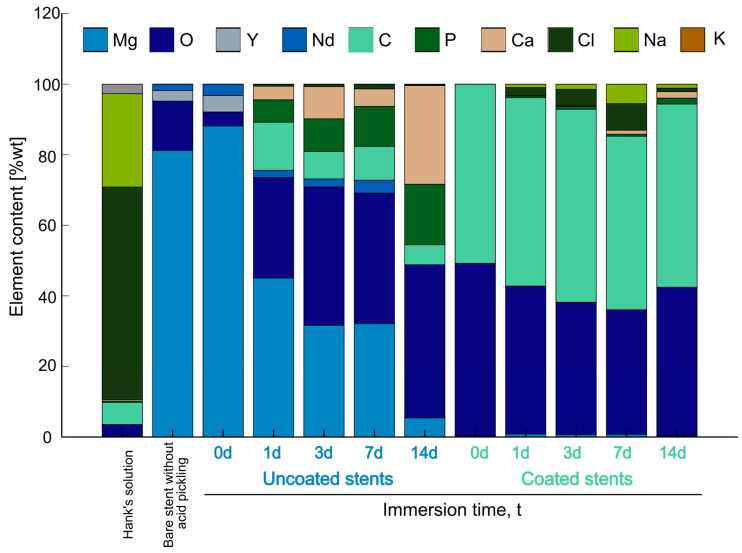
SEM-EDS analysis of Hank’s solution, bare stent without acid pickling, and uncoated and coated stents before and after immersion test.

The SEM-EDS analysis can be used as an indicator of chemical composition to determine the behavior in the immersion tests shown in Figure 7. There is a comparison between Hank’s solution, the WE43 Mg stent without acid pickling, and the uncoated and coated stents before and after the immersion test. Analyzing the composition of Hank’s solution reveals the presence of oxygen (O), carbon (C), phosphorus (P), calcium (Ca), chlorine (Cl), sodium (Na), and potassium (K). It contains carbonate and phosphates, but the majority is NaCl. By analyzing the chemical composition of the bare stent without acid pickling, with the stent indicated as an uncoated stent at 0 days, it can be interpreted that there is an oxidation reduction, but yttrium (Y) and Nd come out from the surface.

Comparing the uncoated stent columns before the immersion test, there is magnesium (Mg), oxygen (O), yttrium (Y), and neodymium (Nd), but the longer the time submerged in degradation, the greater the number of salts formed on the surface. Mg is reduced due to the particles forming on the surface. As can be seen, the primary composition in Hank’s solution is NaCl, but when the stents are uncoated, these elements are not on the surface. Therefore, they are in the solution. According to Zhou et al., since the Cl^−^ ion is small enough, it displaces water molecules from a one-hydration sheath form on the surface of the Mg alloy. This causes Cl^−^ ions to prefer to combine with Mg^2+^ to transform Mg(OH)_2_ into soluble MgCl_2_ [10]. The elements present on the surface are C, P, and Ca. Wang et al. found that Ca and P are concentrated near the surface, whereas the Cl^−^ ions are located deeper, indicating that they are the first to cause corrosion [42]. This may explain the lower Cl content observed in the uncoated stents, as Cl^−^ ions could be more deeply concentrated and thus less detectable at the surface, whereas Ca and P exhibit higher surface concentrations. Corrosion products could be a mixture of Mg(OH)_2_ and Mg carbonate and phosphate [41]. Compared with the results of the coated stents, at 0 days, the composition of the PLA fibers showed C and O on the surface. On other days, it showed Mg, and the corrosion products appeared.

Unlike with the uncoated stents, the ions of Cl and Na adhered to the fibers of the coated stents. For 7 days, a significant amount of corrosion product was on the surface. According to Liu et al., these could be forward and reverse reactions [38].

**Figure 8:**
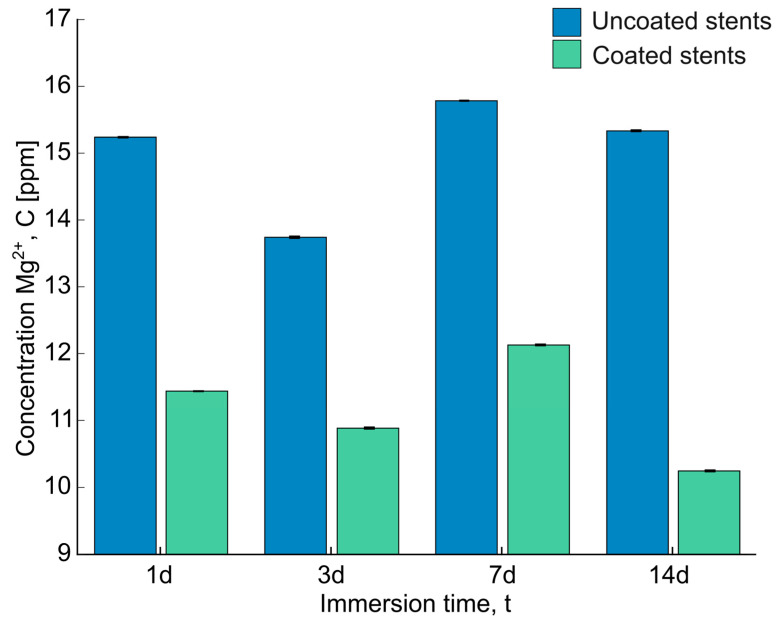
The concentration of Mg^2+^ after being immersed in Hank’s solution for 1d, 3d, 7d, and 14d was examined using an ICP-MS instrument.

The concentration of magnesium ions (Mg^2+^) was analyzed using the ICP-MS instrument shown in Figure 8. The release of magnesium from the uncoated stents ranged between 13.742 ± 0.013 and 15.785 ± 0.010 ppm, with the highest release observed at 7 days. However, the differences in release between days were minimal. In comparison, Xue et al. investigated magnesium release by immersing pure Mg, AZ31, and AZ91 samples in PBS for five days and measuring the release using ICP-MS. They reported significantly different values: 7.679, 8.092, and 7.767 ppm for pure Mg, AZ31, and AZ91, respectively. This discrepancy can be attributed to the differences in the test solutions used for the corrosion experiments and the alloy compositions [41].

On the other hand, a higher magnesium release was observed in the uncoated stents compared to the coated stents, ranging from 10.885 ± 0.012 to 12.129 ± 0.009 ppm, which is due to the protective barrier formed by the PLA fibers. Additionally, as observed in the SEM-EDS analysis, the deposition of corrosion products could decelerate further corrosion. Abdal-hay et al. also observed Ca/P and Ca-P-Mg compound deposition on the substrate, inhibiting further corrosion [16]. The magnesium release trend remained consistent after several days, regardless of whether the stents were coated or uncoated. These results suggest that the PLA fibers protect the magnesium surface, enhancing its integrity over time.

**Figure 9:**
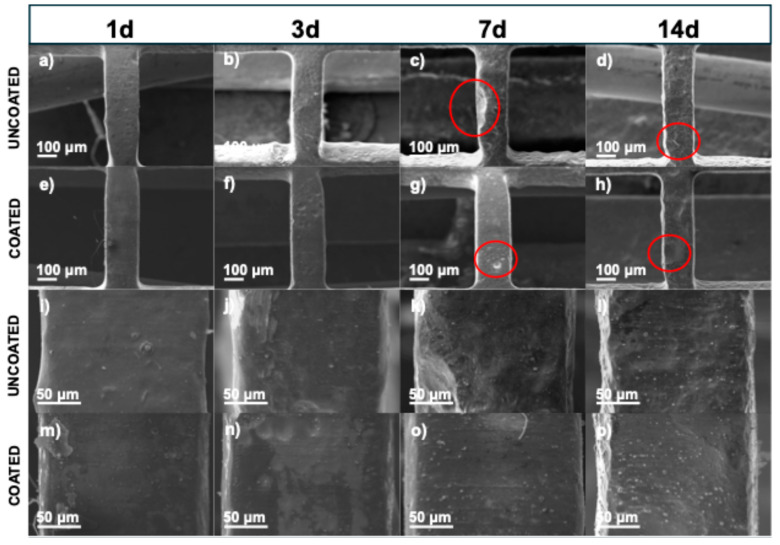
SEM micrographs of stents which were taken after immersion test and after acid cleaning to remove corrosion products: (**a**–**d**) 100× uncoated stents, (**e**–**h**) 100× coated stents, (**i**–**l**) 500× uncoated stents, and (**m**–**p**) 500× coated stents.

**Figure 10:**
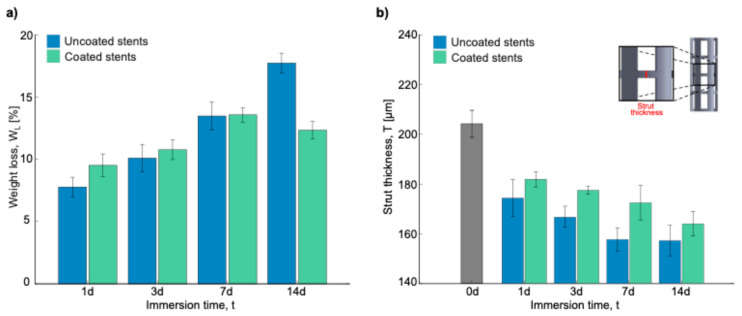
Results after acid cleaning: (**a**) weight loss in percentage of stents, and (**b**) strut thickness.

Figure 9 shows the SEM micrographs of the uncoated and coated stent struts after being cleaned with acid to eliminate corrosion products and better assess the corrosion attack. On the uncoated stents, at 1 day and 3 days, a uniformly corroded surface was observed, while at 7 days and 14 days, many pits could be observed (red circles). Ascencio et al. observed pits with a length of about 2–4 mm on WE43 disks after 5 days of immersion [40]. Compared to the coated stent surfaces, there were fewer and more superficial pits. These results are consistent with those obtained regarding weight loss and strut thickness (Figure 10). In Figure 10a, during the first 7 days, the stents show more significant weight loss when they are coated. However, the uncoated stents have a greater weight loss after longer amounts of time, such as 14 days, with a difference of around 5% compared to the coated stents.

Figure 10b shows the strut thickness measurements to analyze the material lost over the different immersion times. As can be seen, before the degradation process, the strut thickness measurement was 222.68 ± 3.346 μm, a value that was taken as a reference for the uncoated and coated stents. The strut thickness decreased as the days passed in both stent conditions. For the uncoated stents, the strut decrease was 48.39, 55.83, 64.94, and 65.42 μm for 1, 3, 7, and 14 days, respectively. On the other hand, for the coated stents, the decrease was 40.84, 45.13, 50.18, and 58.60 μm for 1, 3, 7, and 14 days, respectively. This is crucial because a structural failure due to corrosion could cause the implanted stent to collapse, disrupting the vascular remodeling process [43]. Based on ASTM-G31, the corrosion rate is calculated using Equation (8) [44]:

(8) $$CR=\frac{K\cdot W}{A\cdot T\cdot D}$$

where CR is the corrosion rate [mm/year], K is a constant [87,600 for mm/year], W is the weight loss [g], A is the exposed area [cm^2^], T is the immersion time [h], and D is the density of the material [g/cm^3^]. The results obtained by taking CR for the 14-day samples were 0.312 ± 0.010 mm/year and 0.216 ± 0.013 for the uncoated and coated stents, respectively. According to these results, there is a reduction in the corrosion rate of ~30%. After performing Welch’s t-test, this difference was found to be statistically significant, with a p-value of 0.002 (n = 3). Although this reduction is promising, statistical confirmation would require a larger sample size. Voicu et al. reported a corrosion rate of 2.03 mm/year for uncoated AZ31 disks, which was reduced to 0.36 mm/year after PLA coating in simulated body fluid (SBF) [27]. Similarly, Kumar et al. presented corrosion rates of different Mg alloys ranging from 0.08 to 1.21 mm/year depending on the surface modification used for cardiovascular stents [45]. Mao et al. demonstrated that MgF_2_-coated JDBM alloys showed a 20% reduction in corrosion rate (0.269 vs. 0.337 mm/year) in artificial plasma [46]. Lewis, G. summarized in a table reductions of 31% to 99.5% in corrosion rates for coated (hydroxyapatite (HA), tricalcium phosphate (TCP), poly(lactic-co-glycolic acid) (PLGA), Ca_2_MgSi_2_O_7_ (akermanite), and poly-L-lactic acid (PLLA)) Mg alloys under immersion tests in physiological media such as phosphate-buffered saline solution (PBS), Hank’s solution, and SBF [47]. Diez et al. combined hydroxyapatite (HA) with poly-L-lactic acid (PLLA) to coat WE43 flat samples. This has shown a reduction in corrosion rate of up to 88% after 10 days in Kokubo SBF [48]. These comparative results reinforce the protective potential of PLA coatings, as observed in this study, particularly for bioresorbable coronary stent applications.

### 3.4. In Vitro Cell Testing

**Figure 11:**
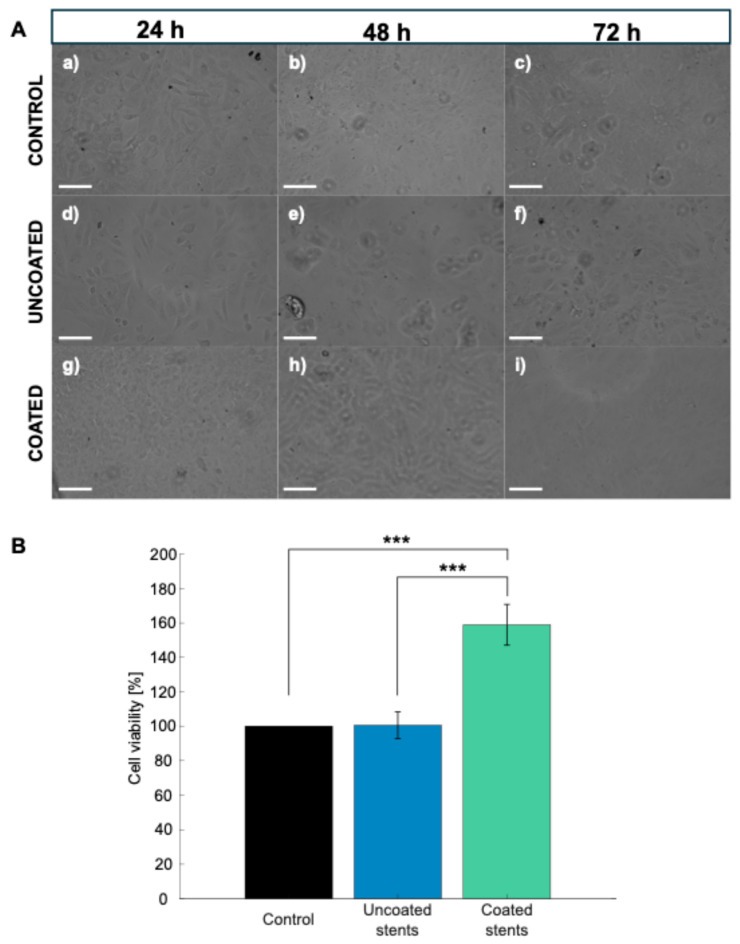
(**A**) Microscopy images of control, uncoated, and coated stents in 12-well plate. Scale bar represents 100 μm: (**a**–**c**) Control samples, (**d**–**f**) uncoated stents, and (**g**–**i**) coated stents. (**B**) Cell viability at 72 h of HDFa cells. Data are presented as mean ± SD (n = 3) (*** *p* < 0.001 Tukey’s test).

Figure 11A shows the morphology images after different exposure times to the tested materials. After 72 h of incubation, the control group cells maintained a fibroblastic morphology and high confluency up to 72 h. The cell density appears lower for the uncoated stents, with signs of stress observed at 72 h. The cell density is higher for the coated stents than the uncoated ones, suggesting that the coating may improve adhesion or viability.

An MTS assay was used to test the cell viability of HDFa cells after 72 h of exposure to the uncoated and coated stents to validate their cytotoxic effects, as shown in Figure 11B. The material is considered nontoxic when the cell viability is above 90% [49]. The cells incubated with the uncoated stents showed a cell viability of 100.48%; meanwhile, the cells incubated with the coated stents showed a greater cell viability, reaching 158.94%. This 58.98% increase in viability could be due to increased proliferation or increased metabolic activity. These results showing a cell viability above 100% have already been reported by Dohle et al., who investigated a biomaterial composed of PDLLA (poly(D,L-lactide)) in various combinations with calcium carbonate (CC), magnesium (Mg), and chitosan (CH). The PDLLA: CC + Mg CH combination achieved a relative HDFa cell viability exceeding 100%, leading them to conclude that this material exhibits good biocompatibility [50]. Statistical analysis revealed significant differences in cell viability among the experimental groups (*p* < 0.000128). In particular, with Tukey’s test, the PLA-coated stents showed a statistically higher cell viability than the uncoated stents and the control group (*p* < 0.00024 and *p* < 0.00023, respectively), indicating the enhanced biocompatibility of the polymeric coating.

Therefore, after reviewing the results related to the evaluation of the cell viability of the HDFa cells, WE43 Mg and PLA were determined to be nontoxic. Furthermore, the PLA coating not only acts as a protective barrier to control stent degradation but may also modulate cellular metabolic activity. The lactic acid released during PLA degradation may serve as an energy source for cells and protect them from oxidative stress [51]. Furthermore, the increase in cell viability observed in the presence of the PLA coating demonstrates that lactic acid stimulates cell proliferation (Figure 11A(c,f,i)). Lampe et al. found an increase in the neural cell population due to a reduction in the intracellular redox state driven by lactic acid release [52].

**Figure 12:**
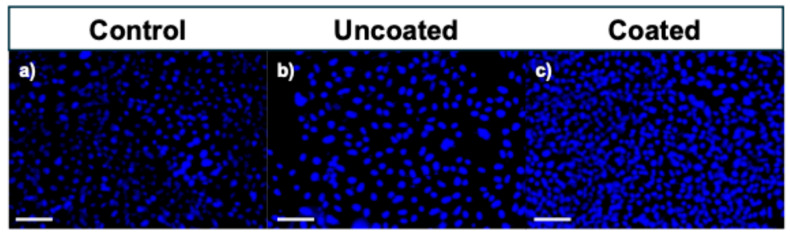
Fluorescence microscopy images of DAPI (cell nuclei)-stained human skin (HDFa) cells after 72 h of incubation. Scale bar represents 100 μm: (**a**) Control sample, (**b**) uncoated stent, and (**c**) coated stent.

Figure 12 shows the fluorescence microscopy DAPI-stained images of the HDFa cells after 72 h of incubation. DAPI staining revealed a higher number of nuclei in the PLA-coated stent environment (Figure 12c) compared to both the uncoated stents and the control group, indicating proliferation. In addition, the cells observed on the PLA-coated surfaces exhibited well-defined, rounded nuclei, suggesting healthy nuclear morphology. In contrast, the cells in the uncoated stent environment (Figure 12b) displayed altered nuclear morphology, potentially reflecting early stages of apoptosis or cellular stress due to the degradative environment of bare magnesium.

**Figure 13:**
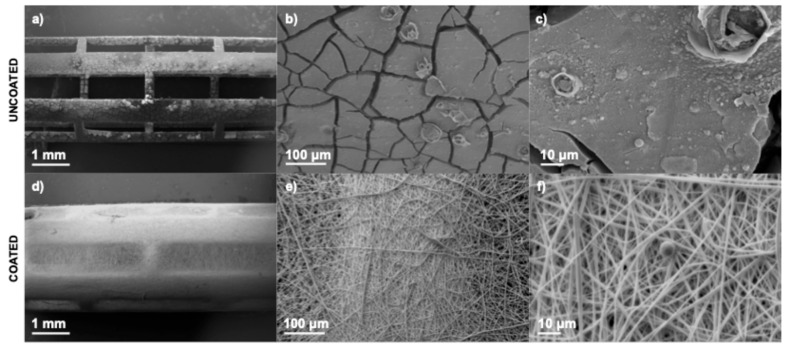
SEM images of stents after 72 h of incubation: Uncoated stent (**a**) 17×, (**b**) 200×, and (**c**) 1000×, and coated stent (**d**) 17×, (**e**) 200×, and (**f**) 1000×.

Figure 13 shows the stents after 72 h of in vitro biological testing. The surface of the uncoated stent (Figure 13a–c) exhibited evident cracking, most likely due to the formation of magnesium corrosion products. Such morphological alterations increase surface roughness, which may adversely affect cellular adhesion and proliferation. In contrast, the stent coated with PLA (Figure 13d–f) preserved its original morphology, indicating that the polymeric layer effectively served as a protective barrier against rapid degradation. Moreover, the presence of a spherical cell between the fibers (Figure 13f) suggests that the coated surface provides a more suitable environment for cellular attachment. These morphological observations align with the viability assay results and support the hypothesis that PLA coatings enhance the biocompatibility of magnesium-based stents by modulating the surface–cell interaction.

## 4. Conclusions

In this study, we provided compelling evidence supporting the use of PLA nanofiber coatings as a protective strategy for WE43 magnesium stents. The key findings are summarized below:

- Stents coated with PLA fibers via electrospinning showed an average fiber diameter of 0.841 ± 0.333 μm and a coating thickness of approximately 23 μm.
- Data on corrosion behavior and magnesium ion release indicated a significant reduction in the degradation rate in coated stents compared to uncoated ones.
- Morphological and chemical composition analyses confirmed the stability of the PLA coating during the immersion period.
- The PLA coating modulates the degradation behavior of the magnesium by regulating the release of Mg^2+^ ions, minimizing drastic pH changes that could compromise cell viability.
- In vitro biocompatibility confirmed that PLA-coated stents are non-toxic, promoting cell adhesion and proliferation, supporting a more favorable environment for vascular healing.

Future research should focus on conducting comprehensive in vivo, biocompatibility, and degradation behavior studies of PLA-coated WE43 magnesium stents under physiological conditions for long-term performance. Additionally, exploring multilayered coatings (i.e., coating both sides of the stents to enhance overall protection), or functionalized coatings (e.g., incorporating anti-inflammatory or pro-healing agents) could further enhance stent performance.

## Data Availability

The data presented in this study are available upon reasonable request from the corresponding author.

## Supplementary Materials

The following supporting information can be downloaded at: https://www.mdpi.com/article/10.3390/polym17111510/s1, Figure S1: Crack densities: (**a**–**d**) SEM micrographs showing sections of the stent strut after 1, 3, 7, and 14 days of immersion, respectively, and (**e**–**h**) corresponding binary images generated through threshold analysis. Scale bar 50 μm.

## Abbreviations

The following abbreviations are used in this manuscript:

Mg	Magnesium
PLA	Polylactic acid
CLF	Chloroform
AC	Acetone
SEM	Scanning electron microscope
EDS	Energy-dispersive spectrometer
HDFa	Human dermal fibroblasts
DMEM	Dulbecco’s Modified Eagle’s Medium
FBS	Fetal bovine serum
PBS	Phosphate-buffered saline
CR	Corrosion rate
