# Supplementary figures and images for: Modulated Degradation of Polylactic Acid Electrospun Coating on WE43 Stents
Source: Polymers (Basel). 2025 May 28;17(11):1510. doi: 10.3390/polym17111510 (PMC12157572; doi:10.3390/polym17111510)

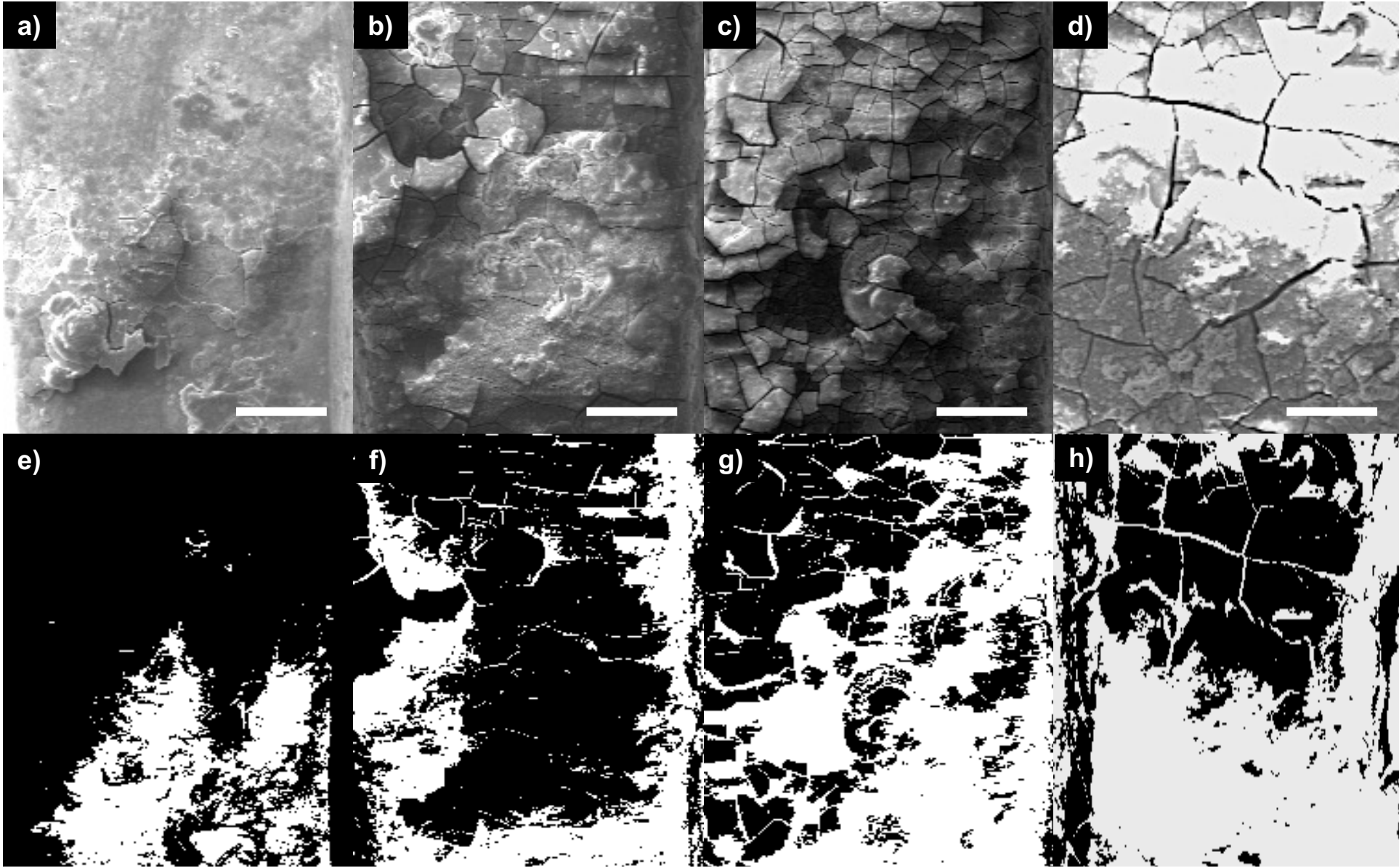

Supplement: Supplementary file 1 [file polymers-17-01510-s001.zip › Figure S1.pdf]
